# Formulation and Characterization of Deep Eutectic Solvents and Potential Application in Recycling Packaging Laminates

**DOI:** 10.3390/polym16192781

**Published:** 2024-09-30

**Authors:** Adamantini Loukodimou, Christopher Lovell, Tianmiao Li, George Theodosopoulos, Kranthi Kumar Maniam, Shiladitya Paul

**Affiliations:** 1 Materials Innovation Centre, School of Engineering, University of Leicester, Leicester LE1 7RH, UK; 2 Materials Performance and Integrity Technology Group, TWI Technology and Training Centre–North East, Middlesbrough, Cleveland TS2 1DJ, UK; 3 Materials Performance and Integrity Technology Group, TWI Ltd., Cambridge CB21 6AL, UK

**Keywords:** DES, laminate packaging materials, blister packs, recycle, carboxylic acids, delamination

## Abstract

Deep Eutectic Solvents (DESs) show promising abilities for the delamination of multilayer packaging films that are used in food packaging and in pharmaceutical blister packs. Due to the complexity of their structure, the recycling of such materials is a challenging task, leading to the easiest or cheapest disposal option of either landfill or incineration. Towards the development of ‘green’ solvents for efficient waste management and recycling, this research focuses on the preparation of a range of hydrophobic and hydrophilic DESs based on carboxylic acids in combination with various naturally derived aliphatic and aromatic organic compounds as well as amino acids. Chemical and physical characterization of the solvents was undertaken using differential scanning calorimetry, rheometry, and density measurements for the determination of their properties. Subsequently, batches of solvent were tested against different types of consumer packaging to evaluate the ability of the DES to delaminate these structures into their component materials. The laminate packaging waste products tested were Al/PE, PE/Al/PET, Al/PE/paper, and PVC/PE/Al. Separated films were collected and studied to further examine the effect of solvent delamination on the materials. Depending on the DES formulation, the results showed either partial or full delamination of one or more of the packaging materials, albeit there were challenges for certain solvent systems in the context of delivering a broad delamination efficiency. Variables including temperature, agitation rate, mixing time, and solvent ratios were investigated via a Design of Experiments process to assess the effects of these parameters on the delamination outcome. The results showed that the DESs presented in this research can offer an efficient, low-energy, affordable, and green option for the delamination of laminate packaging materials.

## 1. Introduction

Multilayer packaging is widely used due to its functionality and convenience, and its demand has been increasing in recent years [1]. At the same time, due to the heterogeneity of the multilayer structure, the challenges of the recycling of multilayer packaging materials have become issues that cannot be ignored [2]. The current commercial recycling systems were developed primarily for sorting and recycling mono-material waste streams [1] and, as such, multilayer packaging is still classified as non-recyclable waste [3]. The main treatment methods for this type of packaging waste remain incineration and landfill [4]. In this regard, the recycling of multilayer packaging has received increased attention and research in recent years.

The main recycling processes studied can be divided into three categories. One approach is to use compatibilizers in the mechanical recycling process to increase the compatibility between different polymers in each layer [5] so that the recycled product can be used as a homogeneous material for remanufacturing. Another process is selective dissolution–precipitation based on the mechanism that different polymers have different solubility in different solvents and under different conditions. In this instance, the target polymers in multilayer packaging waste can be separated through a series of dissolution–precipitation processes [4]. The third methodology is delamination of the multilayer structure. By dissolving or decomposing the tie layer used to bond the polymers and/or non-polymeric materials in each layer, the separating of the target components in the multilayer packaging is achieved [2]. The use of compatibilizers is normally expensive and complicated [6], and the recycled product will lose the purity of the original polymer [7]. The process of selective dissolution–precipitation usually involves multiple steps and the use of multiple solvents. Compared with the above two recycling methods, delamination has the advantages of a simple process, requiring fewer solvent types for a wide range of materials and separation of the components without alternation of the polymer structure. Therefore, it is a very promising recycling method for complex multi-material laminates [2].

For the delamination process, the diffusion of the solvent in the polymers and the solubility of the tie layer polymer in the solvent are the main factors determining the delamination rate [8]. Therefore, almost all studies on the delamination process mentioned the pretreatment of cutting multilayer packaging waste into small pieces before reactions [2,8,9,10]. In recent years, various solvents have been used to study the delamination of multilayer packaging waste. These solvents mainly include inorganic acids (nitric acid [9]), organic acids (carboxylic acids [2,8] and their mixtures with other organic components [10]), and other organic solvents (such as acetone [11], ethylene glycol oligomers [12], etc.). Studies have found that carboxylic acid solvents have a wide range of applicability for the delamination of various multilayer structures [2]. Small-molecule carboxylic acids are considered to be very promising delamination solvents because of their fast diffusion rate through the polymer layers of multilayer packaging waste [13]. In 2021, Ügdüler et al. [2] experimentally confirmed that formic acid can effectively and efficiently delaminate PET-PE-based multilayer packaging by dissolving the PU tie layer. In 2020, Nieminen et al. [10] reported a process in which lactic acid, as a green solvent, dissolves acrylic-based adhesive to effectively delaminate and recycle PVC and aluminum from pharmaceutical blister packaging waste. It is worth mentioning that the authors also tested the delamination effect of lactic acid–choline chloride, a deep eutectic solvent (DES) system, on the recycling of pharmaceutical blister packaging. Their work has opened up a new area for the selection and design of delamination solvents for the recycling of multilayer packaging waste.

A DES is defined as a mixture containing a hydrogen bond acceptor (HBA) and a hydrogen bond donor (HBD) [14,15]. Due to the formation of a complex hydrogen bond network between the two components (HBA and HBD), the melting point of DES is much lower than the melting points of each component [16], and some DESs are even stable liquids at room temperature [17]. DESs are normally regarded as non-toxic, non-flammable, chemically stable, biocompatible, biodegradable, simple to prepare, and low-cost [18,19,20]. Most of the components of DES can be obtained from natural materials. Therefore, DESs are in line with the development goals of green solvents and are highly favored with great potential in many application fields such as extraction, chemical synthesis, purification, battery technologies, etc. [21]. Typical HBAs are quaternary ammonium salts (such as choline chloride), and typical HBDs include polyols and acids and amines [15]. More recently, hydrophobic DES systems have also been described in the literature, which combine carboxylic acids or alcohols with long-chain alkyl acids, fatty acids of different chain lengths, and terpenoids with carboxylic acids [14].

Based on a background investigation of the recycling of multilayer packaging and the application of DESs, we found that carboxylic acid-based DESs have great potential to be applied to the delamination of multilayer packaging waste. However, there are very limited studies or reports specifically on the use of DES for packaging recycling [10]. In this study, we have developed new DES systems based on amino acids, an aliphatic diol, and terpenoids in combination with a carboxylic acid to produce a range of DESs with hydrophilic and hydrophobic characters that are suitable for the delamination of multilayer packaging. We have used examples of packaging laminate waste comprising PE, PET, PVC, paper, and aluminum to test the efficacy of the solvents. Further, additional studies were carried out on selected systems to investigate the effect of the reaction conditions, temperature, time, and stirring rate on the success of delamination via a Design of Experiments methodology.

## 2. Materials and Methods

### 2.1. Solvents

Chemicals acquired from Merck Life Sciences UK Ltd., Gillingham, UK, were used without further purification, including acetic acid (≥99%), carvacrol (≥98%), guaiacol (≥99%), eugenol (≥98%), thymol (≥99%), propylene glycol (≥99.5%), betaine (≥98%), and L-proline (≥99%).

In total, seven DES formulations were prepared according to the molar ratios presented in Table 1. Molar quantities of the chemical components were weighed into glass containers before being heated to 70 °C and stirred until a homogenous clear liquid was formed using a VELP AREX6 Digital Pro Hotplate Stirrer (Heidolph Instruments GmbH, Schwabach, Germany).

Characterizations of the solvents included a visual assessment of water miscibility, measurements of density and viscosity, and measurement of the crystallization temperature.

Density measurements were performed using an Elcometer 1800 Density Cup stainless steel pycnometer (Elcometer Ltd., Manchester, UK). The filled pycnometer was held for 1 h in a Binder MKFY 115 Dynamic Climate Chamber (Tuttlingen, Germany) temperature and a humidity-controlled oven at 20.0 ± 0.5 °C at 50 ± 1% RH prior to mass measurements using Ohaus Pioneer balance (2 d.p.).

Room temperature measurements of viscosity were performed at a shear rate of 10 s^−1^ using Bohlin Gemini Nano HR rheometer (Malvern Panalytical, Worcestershire, UK) equipped with 40 mm diameter parallel plates at a gap of 200 μm. Shear rate ramp tests between 0.1 and 100 s^−1^ confirmed the Newtonian behavior of all the solvents.

### 2.2. Packaging Films and Blister Packaging

Three types of multilayer packaging films and one example of pharmaceutical blister packaging were supplied by Plastigram Industries A.S. (Tovární, Czechia) and Mikrolin Hungary Kft (Tatabánya, Hungary). All materials were sourced from primary waste streams.

**Figure 1:**
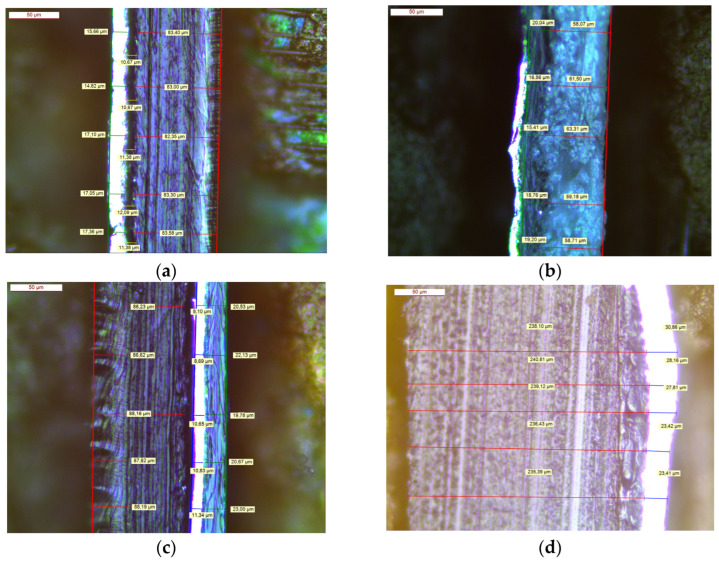
Cross-section images of samples (**a**) PE/Al, (**b**) Al/PE/Paper, (**c**) PE/Al/PET, and (**d**) blister sample consisting of PVC/lidding film/Al/ink.

Figure 1 presents optical micrograph images which detail the structures of the three laminate packaging films, which included PE/Al (Figure 1a), Al/PE/Paper (Figure 1b), and PE/Al/PET (Figure 1c), and an example of the pharmaceutical blister pack with the structure, PVC/lidding film/Al/ink (Figure 1d). A summary of the thickness for the various plastic and aluminum components is given in Table 2.

### 2.3. Delamination

#### 2.3.1. Screening Tests

A total of 30 ml of solvent was added to a 125 mL glass bottle and heated to 70 °C under stirring using a VELP AREX6 Digital Pro Hotplate Stirrer equipped with a VTF Temperature Probe and allowed to equilibrate at temperature.

Samples of each packaging laminate film were cut into (approx.) square pieces of 10 mm dimension. Ten pieces of each film (~0.3 g or ~0.6% *w*/*w*) were then immersed in the 30 ml of solvent under stirring conditions at temperature. The delamination period was set at 45 min, after which the bottle was removed from the hotplate stirrer and the contents were poured into a Büchner funnel to separate the film pieces from the solvent. The film pieces were then briefly rinsed with isopropyl alcohol to dilute and facilitate the evaporation of any residual solvent coating the surfaces. The materials were then allowed to dry under ambient conditions while stored in a fume cupboard.

The test was then repeated for each solvent.

#### 2.3.2. Design of Experiments

A series of experiments was performed according to a Design of Experiments matrix in order to evaluate the effects of temperature (A), time (B), and stirrer speed (C) on delamination. Initially, five trials were performed according to a 2-level factorial screening design (2^3-1^_III_) with a center point. Following these experiments, the design was augmented with six axial design points akin to a central composite design (α = 1.68), thus expanding the design space. The corresponding parameter values for each factor are recorded in Table 3.

**Figure 2:**
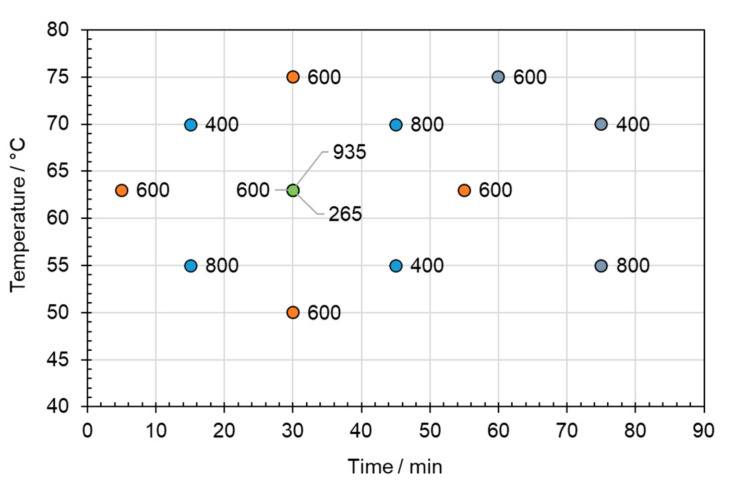
Combinations of temperature, time, and stirrer speed (rpm) (denoted by labels) used in the experimental design.

Finally, three additional experiments were performed at longer durations whilst preserving the symmetry of the original design: one for 60 min at 75 °C and two for 75 min at 55 °C and 70 °C. The array of experimental conditions is shown in Figure 2.

Thymol/acetic acid and betaine/acetic acid solvent systems were selected for the trials, which were undertaken on the PE/Al, Al/PE/paper, and PE/Al/PET packaging laminates only.

### 2.4. DSC

Experiments on the solvents and polymer films were performed using a DSC 214 Polyma (Netzsch Thermal Instruments UK Ltd., Wolverhampton, UK) under an inert atmosphere of N_2_ purge gas (40 mL min^−1^). Calibrations of the instrument temperature and heat flow were made using high-purity standards of indium (99.999%), tin (99.999%), bismuth (99.999%), zinc (99.999%), and cesium chloride (99.999%).

Small quantities of each sample type were placed in aluminum pans with masses in the range of 9–13 mg. For solvents, tests were performed via a temperature ramp from 25 to −80 °C at 10 °C min^−1^ to determine crystallization temperature whilst tests on plastic films were performed via a temperature ramp from 25 to 300 °C at 10 °C min^−1^ to determine glass transition and melting temperatures.

### 2.5. FTIR

In the case of packaging materials, adhesives and inks also used in the assembly of the laminate structures will contribute to FTIR spectra if they remain on the surface of the PE, PET, and aluminum foils. An Alpha Platinum ATR FTIR Spectrometer (Bruker Corporation, Massachusetts, USA) was used to evaluate the separated plastics and aluminum. The number of scans for each measurement was 32 with a scan range of 4000–400 cm^−1^ at a resolution of 4 cm^−1^. Spectra were taken at three different sample locations. The spectra obtained from the three different points were in good agreement; hence, one of them is presented in the results. The data were collected and later processed in MS Excel.

## 3. Results and Discussion

### 3.1. Delamination

#### 3.1.1. Screening Experiments

**Figure 3:**
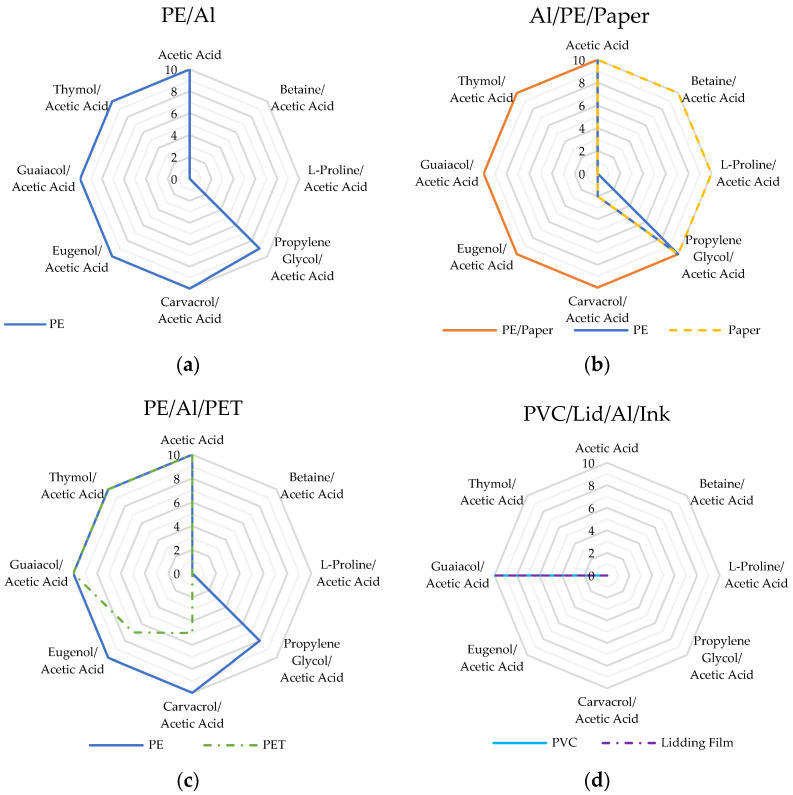
Radial plots illustrate the results of the screening trials showing the delamination count (out of 10) for each of the packaging materials: (**a**) PE/Al; (**b**) Al/PE/paper; (**c**) PE/Al/PET; and (**d**) PVC/lidding film/Al/ink.

The results from screening experiments to assess the efficacy of the solvent systems in terms of their ability to delaminate the three packaging multilayers are recorded in Table 4. The values in the table represent the delamination count (out of 10) for the number of pieces of film that were separated completely during the tests at 70 °C after a period of 45 min. These results are also visualized in Figure 3, which displays radial plots of the delamination count against each solvent for each packaging material.

Acetic acid alone was found to be a very effective solvent for the delamination of the three packaging materials achieving the complete separation of PE/Al, Al/PE/paper, and PE/Al/PET [8]. In contrast, the hydrophilic amino acid-based solvents, betaine/acetic acid and L-proline/acetic acid, proved only to be efficient in the separation of paper from Al/PE/paper and did not result in any complete film separations from either PE/Al or PE/Al/PET. Propylene glycol/acetic acid resulted in the complete delamination of Al/PE/paper laminate. The hydrophobic solvent systems, carvacrol/acetic acid, eugenol/acetic acid, guaiacol/acetic acid, and thymol/acetic acid, as well as the hydrophilic propylene glycol/acetic acid solvent, all showed promising results in terms of a high degree of delamination for PE from PE/Al and PE/Al/PET and PE/paper from Al/PE/paper. The hydrophobic systems also proved effective in the separation and de-inking of the reverse-printed PET layer from PE/Al/PET.

Only guaiacol/acetic acid yielded successful delamination of the blister packaging laminate. Very significant swelling of PVC was evident at the conclusion of the test, and this was considered the driving mechanism for the separation of the layers.

Following the screening tests, a more detailed study of the dependence of the packaging laminate delamination on the trial conditions was carried out with two selected solvents, specifically thymol/acetic acid and betaine/acetic acid. The choice of thymol/acetic acid reflected the excellent delamination performance in the screening trials at 70 °C for 45 min across the packaging laminates, as presented above in Table 4 and Figure 3. In contrast, betaine/acetic acid resulted only in the delamination of paper from Al/PE/paper, and in this context, it did not perform well compared to others. However, as an example of an amino acid-based solvent system with extremely promising ‘green’ credentials, it was decided that it would be of value to establish whether or not successful delamination could be achieved with all components of the test materials under different conditions, in particular longer durations.

#### 3.1.2. Design of Experiments

A series of delamination experiments under different conditions (according to the Design of Experiments matrix shown in Figure 2) was performed using thymol/acetic acid and betaine/acetic acid.

The results of the trials were subsequently analyzed using Design Expert V13 software (StatEase^®^, Inc., Minneapolis, MN, USA). A linear model based on the Logit function was fit to the datasets (Equation (1)):

F(p_i_) = ∑_i_ ln (p_i_/(1 − p_i_)) = ∑_i_ β_A_ × A_i_ + β_B_ ∙ B_i_ + β_C_ ∙ C_i_ + ε_i_ (1)

where the probability of delamination, p_i_, for a given set of experimental conditions, i, was defined as equal to the fraction of the delamination counts out of 100. The software employed logistic regression (maximum likelihood estimation) to evaluate the significance of the model coefficients, β_f_, for each factor, f.

#### 3.1.3. Thymol/Acetic Acid

The results of the delamination experiments with thymol/acetic acid are presented in Table 5. As seen from the delamination counts, a wide range of temperature and time conditions (within the experimental design space) were effective in achieving separation at the PE/Al interface. A narrower set of experimental conditions resulted in the separation of PET from Al in PE/Al/PET with successful delamination observed at temperatures and times above 63 °C and 30 min. In the case of Al/PE/paper, albeit PE/paper was readily separated from Al, the combined PE/paper layers remained largely intact for all conditions, with only partial separation of the components. However, compared to PE/Al and PE/Al/PET structures, PE formed only a minor component of the Al/PE/paper laminate at 18 ± 2 μm thickness (Table 2).

**Figure 4:**
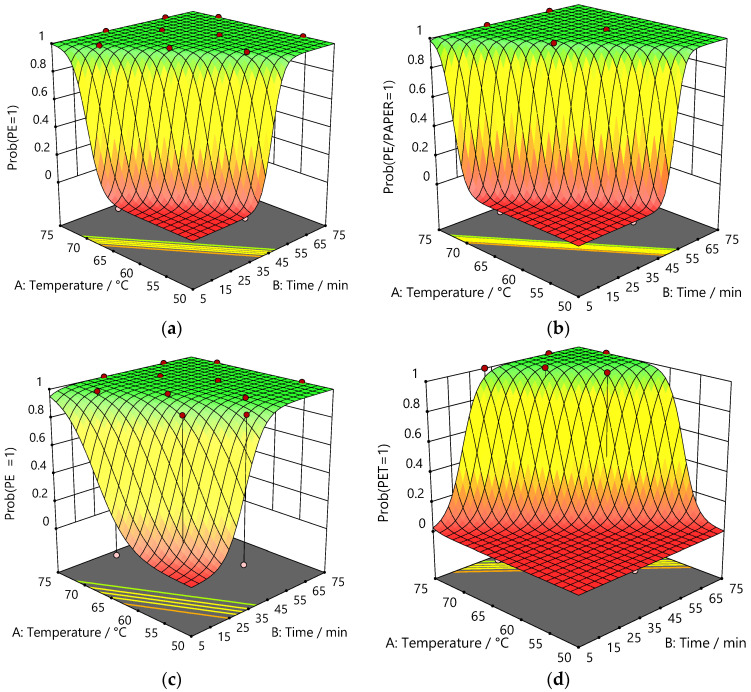
Plots comparing the probability of delamination as a function temperature and time at 600 rpm for (**a**) PE from PE/Al; (**b**) PE/paper from Al/PE/paper; (**c**) PE from PE/Al/PET; and (**d**) PET from PE/Al/PET using thymol/acetic acid.

To better understand the relationship between delamination and the conditions of temperature, time, and stirrer speed, it was important to perform a detailed evaluation of the datasets. The outcomes of the logistic regression analyses are presented in Table 6, where significant terms of the models (Equation (1)) fit to the delamination counts are indicated by *p* < 0.05. Correspondingly, the calculated coefficients of the model for each significant factor were then used to generate surface plots, as shown in Figure 4, which convey the dependence of the probability of delamination for PE, PE/paper, and PET on temperature and time at a stirrer speed of 600 rpm for thymol/acetic acid. The plots illustrate transitions from the negligible probability of successful delamination at lower temperatures and shorter periods of time to the high probability of complete delamination at higher temperatures and longer times. In general, the separation of PE layers at the PE/Al interfaces occurred more readily than the separation of the reverse-printed PET layer at the Al/PET interface.

#### 3.1.4. Betaine/Acetic Acid

In Table 7, the delamination counts for experiments performed with betaine/acetic acid are shown. In this instance, the solvent proved to only be effective for the separation of paper from Al/PE/paper, even when extending the duration to 75 min at 70 °C.

**Figure 5:**
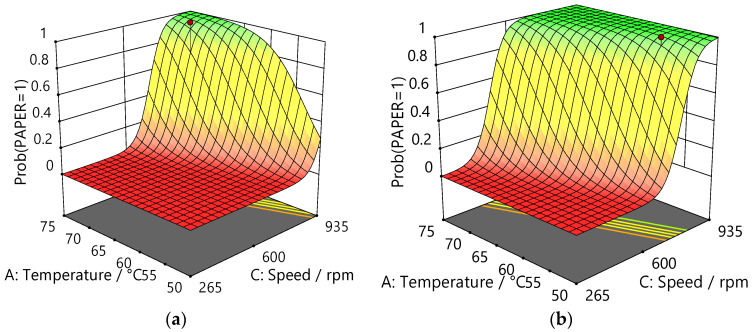
Plots comparing the probability of the delamination of paper from Al/PE/paper as a function of temperature and stirrer speed: (**a**) at 45 min and (**b**) at 75 min using betaine/acetic acid.

The result of the regression analysis applied to the delamination of paper is presented in Table 8, and surface plots showing the dependence of the probability of delamination on temperature and stirrer speed at 45 min and 75 min time points are shown in Figure 5. All three factors, temperature, time, and stirrer speed, were found to impact the delamination of paper.

### 3.2. FTIR and DSC Analysis of Delaminated Films

FTIR and DSC analyses were carried out on examples of delaminated and dried film samples in order to assess the condition of materials.

**Figure 6:**
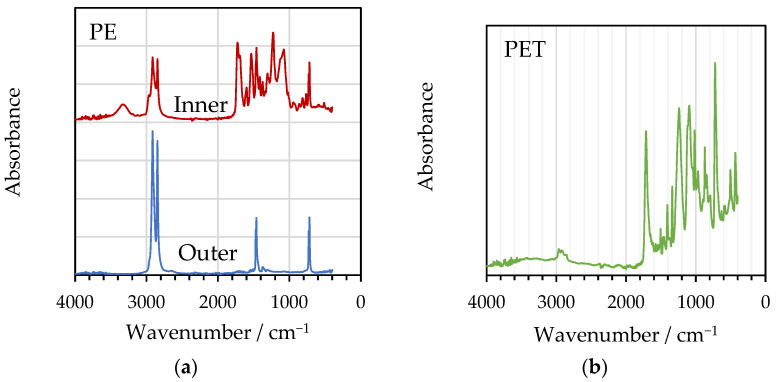
FTIR spectra obtained from delaminated films: inner and outer surfaces of (**a**) PE and (**b**) PET delaminated with thymol/acetic.

Figure 6 presents the FTIR spectra obtained from PE and PET films separated using thymol/acetic acid. The separated PE films obtained from PE/Al and PE/Al/PET were found to exhibit different spectra when comparing the inner and outer surfaces of the films. This was due to residual adhesive on the inner surface, which dominated the spectrum. The spectrum from the outer surface was consistent with library spectra poly(ethylene) and showed no signs of oxidative degradation, which might be indicated by the appearance of a carbonyl band at ~1720 cm^−1^. Similarly, the spectra obtained for PET were consistent with library spectra.

**Figure 7:**
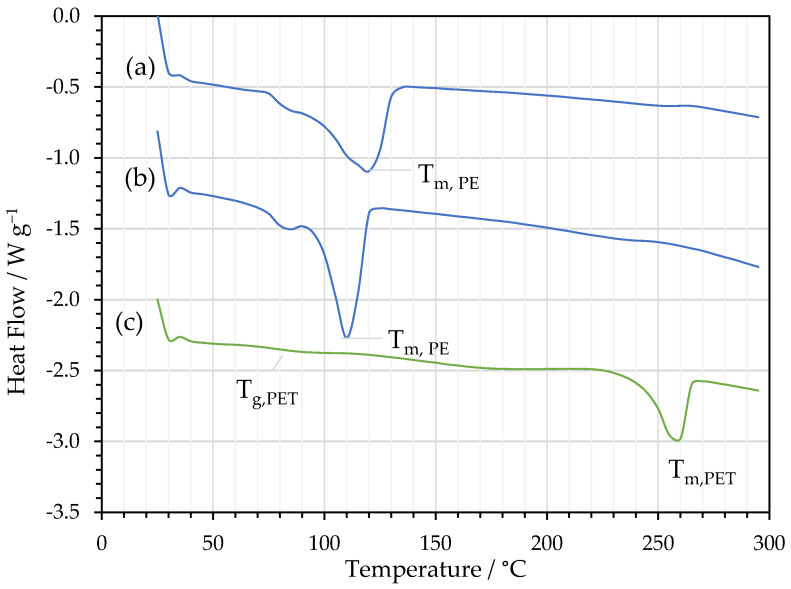
DSC thermograms obtained from delaminated films: (**a**) PE from PE/Al; (**b**) PE from Al/PE/paper; and (**c**) PET from PE/Al/PET.

Examples from the thermal analysis of delaminated PE and PET separated using thymol/acetic acid are shown in Figure 7. A summary of parameters from the analysis of the DSC thermograms is presented in Table 9.

DSC traces obtained for PE films from PE/Al and Al/PE/paper in Figure 7a,b show typical broad melting endotherms of poly(ethylene) with peak melting temperatures (T_m_) of 118 °C and 111 °C, respectively. The integrated peak areas yielded enthalpies of fusion (ΔH_f_) of 103 J g^−1^ and 97.4 J g^−1^, which were then used to calculate crystallinities (χ_c_) of 35% and 33% (assuming a literature value of 290 J g^−1^ for the fusion of purely crystalline linear poly(ethylene)).

The glass transition temperature and melting temperature for PET were 78 °C and 257 °C, respectively, with an enthalpy of fusion of 38.2 J g^−1^, which corresponded to a crystallinity of 27% (assuming a literature value of 140 J g^−1^ for the fusion of purely crystalline linear poly(ethylene terephthalate)).

The thermal analysis of PE and PET films suggests that the semi-crystalline morphologies of the polymers were not unduly influenced by the solvent treatment as the parameter values which characterized the melting and glass transition behaviors are considered typical of these materials. It is also noted that neither the FTIR nor the DSC traces indicated significant amounts of residual solvent within the dried films as there were no noteworthy anomalous features, for example, endothermic events that might occur due to the volatilization of solvents on heating in the case of DSC. These results are encouraging in the context of the quality of delaminated materials obtained and their potential for future recycling and re-use.

## 4. Conclusions

In this research, a diverse range DESs which combine naturally derived hydrophilic and hydrophobic H-bond acceptors with acetic acid, a H-bond donor, have been investigated as novel solvent systems for the delamination and recovery of component materials from multilayer plastic packaging.

Three packaging laminate structures were tested in the study: PE/Al, Al/PE/paper, and PE/Al/PET. Through screening studies at a temperature of 70 °C, hydrophobic DESs, which included carvacrol/acetic acid, eugenol/acetic acid, guaiacol/acetic acid, and thymol/acetic acid, were shown to effectively delaminate PE and PET from the laminate structures. Propylene glycol/acetic acid was also similarly effective in achieving the separation of PE at the PE/Al interface and to a lesser extent PET. The hydrophilic DES based on the amino acids betaine and L-proline were only effective in the separation of paper from the Al/PE/paper structure. With regard to the blister pack, guaiacol/acetic acid was used to successfully delaminate PVC, albeit that significant swelling of the plastic was noted immediately upon the extraction of the material.

Subsequently, the effects of the test on conditions on efficacy and efficiency of delamination were assessed for thymol/acetic acid and betaine/acetic acid with PE/Al, Al/PE/paper, and PE/Al/PET films. The results demonstrated that these complex multilayer packaging laminates could be readily separated into the component plastics, paper, and aluminum over a range of conditions and that models fit to data allowed us to learn about the time, temperature, and stirrer speed dependences of our small laboratory-scale process.

The success of the hydrophilic and hydrophobic DES systems in achieving delamination demonstrates that this technology could be further developed and explored as a potential ‘green’ solution to the issue of recycling complex multilayer laminate packaging and, thereby, facilitate the recycling and re-use of these valuable materials in our economy.

## Figures and Tables

**Table 1:** Solvents and molar ratios.

Solvent	Molar Ratio	Water Miscibility	Density/g cm^−3^	Viscosity/mPa s	Crystallization Temperature/°C
Acetic Acid	-	miscible	1.049	2.4 ± 0.6	−16.7
Betaine/Acetic acid	1:4	miscible	1.118	41.7 ± 0.7	Not detected
L-Proline/Acetic Acid	1:3	miscible	1.166	117 ± 2	Not detected
Propylene Glycol/Acetic Acid	1:2	miscible	1.043	16.0 ± 0.5	Not detected
Carvacrol/Acetic Acid	1:1	immiscible	0.993	6.9 ± 0.5	Not detected
Eugenol/Acetic Acid	1:1	immiscible	1.061	5.0 ± 0.4	Not detected
Guaiacol/Acetic Acid	1:1	immiscible	1.107	4.0 ± 0.8	Not detected
Thymol/Acetic Acid	1:1	immiscible	0.991	7.0 ± 0.5	Not detected

**Table 2:** Thickness of component films.

Material	Film Thickness/μm				
	Al	PVC	PE	PET	Paper
PE/Al	16 ± 1	-	83 ± 1	-	-
Al/PE/Paper	10 ± 2	-	18 ± 2	-	58 ± 3
PE/Al/PET	10 ± 1	-	87 ± 1	21 ± 1	-
Blister Pack	26 ± 1	211 ± 1	-	-	-

**Table 3:** Factors and parameter values used in the experimental design.

	Factor	Unit	Low	High	Centre	−α	+α
A	Temperature	°C	55	70	63	50	75
B	Time	min	15	45	30	5	55
C	Stirrer Speed	rpm	400	800	600	265	935

**Table 4:** This table records the delamination count (out of 10) for the component films from screening experiments with different solvent systems.

Solvent	Molar Ratio	PE/Al	Al/PE/Paper			PE/Al/PET		Blister Pack	
		PE	PE/Paper	PE	Paper	PE	PET	PVC	Lidding Film
Acetic Acid	-	10	10*^a^*	10	10	10	10	0	0
Betaine/Acetic Acid	1:4	0	0	0	10	0	0	0	0
L-Proline/Acetic Acid	1:3	0	0	0	10	0	0	0	0
Propylene Glycol/Acetic Acid	1:2	9	10*^a^*	10	10	8	0	0	0
Carvacrol/Acetic Acid	1:1	10	10*^a^*	2	2	10	5	0	0
Eugenol/Acetic Acid	1:1	10	10	0	0	10	7	0	0
Guaiacol/Acetic Acid	1:1	10	10	0	0	10	10	10	10
Thymol/Acetic Acid	1:1	10	10	0	0	10	10	0	0

*^a^* PE/paper fully delaminated prior to separation of PE and paper.

**Table 5:** This table records the delamination count (out of 10) for the component films from experiments performed under different conditions with thymol/acetic acid.

#	Conditions			PE/Al	Al/PE/Paper			PE/Al/PET	
	A: Temperature /°C	B: Time /min	C: Speed /rpm	PE	PE/Paper	PE	Paper	PE	PET
1	55	45	400	10	0	0	0	10	0
2	70	15	400	10	0	0	0	10	0
3	55	15	800	0	0	0	0	5	0
4	70	45	800	10	10	0	0	10	10
5	63	30	600	10	10	0	0	10	0
6	63	55	600	10	10*^a^*	2	2	10	7
7	75	30	600	10	10*^a^*	0	0	10	8
8	50	30	600	0	0	0	0	3	0
9	63	5	600	0	0	0	0	0	0
10	63	30	600	10	10	0	0	10	0
11	63	30	265	10	0	0	0	10	0
12	63	30	935	10	10	0	0	10	0
13	75	60	600	10	10	0	0	10	10
14	70	75	400	10	10	0	0	10	10
15	55	75	800	10	10*^a^*	1	1	10	0

*^a^* PE/paper fully delaminated prior to separation of PE and paper.

**Table 6:** Summary of significant factors in the linear models for the delamination of PE from PE/Al/, PE/paper from Al/PE/paper, and PE and PET from PE/Al/PET using thymol/acetic acid.

		Factor* p*-Values			
Material	Film	Model	A: Temperature (°C)	B: Time (min)	C: Stirrer Speed (rpm)
PE/Al	PE	<0.0001	<0.0001	<0.0001	n/a
Al/PE/Paper	PE/Paper	<0.0001	<0.0001	<0.0001	<0.0001
PE/Al/PET	PE	<0.0001	<0.0001	<0.0001	0.0006
	PET	<0.0001	<0.0001	<0.0001	0.0192

**Table 7:** This table records the delamination count (out of 10) for the component films from experiments performed under different conditions with betaine/acetic acid.

#	Conditions			PE/Al	Al/PE/Paper			PE/Al/PET	
	A: Temperature /°C	B: Time /min	C: Speed /rpm	PE	PE/Paper	PE	Paper	PE	PET
1	70	45	800	0	0	0	10	0	0
2	55	45	400	0	0	0	0	0	0
3	63	30	600	0	0	0	0	0	0
4	70	15	400	0	0	0	0	0	0
5	75	30	600	0	0	0	1	0	0
6	63	55	600	0	0	0	0	0	0
7	55	75	800	0	0	0	10	0	0
8	75	60	600	0	0	0	3	0	0
9	70	75	400	0	0	0	0	0	0
10	55	15	800	0	0	0	0	0	0
11	70	15	800	0	0	0	0	0	0
12	70	45	800	0	0	0	10	0	0
13	63	30	935	0	0	0	3	0	0
14	63	30	265	0	0	0	0	0	0
15	50	30	600	0	0	0	0	0	0

**Table 8:** Summary of significant factors in the linear models for the delamination of paper from Al/PE/paper using betaine/acetic acid.

		Factor* p*-Values			
Material	Film	Model	A: Temperature (°C)	B: Time (min)	C: Stirrer Speed (rpm)
Al/PE/Paper	Paper	<0.0001	0.0018	<0.0001	<0.0001

**Table 9:** Summary of parameters from the analysis of the DSC thermograms.

Material	Film	DSC Parameters			
		T_g_ /°C	T_m_ /°C	ΔH_f_ /J g^−1^	χ_c_ /%
PE/Al	PE	-	118	103	35
Al/PE/Paper	PE	-	111	97.4	33
PE/Al/PET	PET	78	257	38.2	27

## Data Availability

The original contributions presented in the study are included in the article; further inquiries can be directed to the corresponding author.
